# Feasibility, Fidelity and Acceptability of a Person‐Centred Care Transition Support Intervention for Stroke Survivors: A Non‐Randomised Controlled Study

**DOI:** 10.1111/hex.70057

**Published:** 2024-10-07

**Authors:** Sebastian Lindblom, Maria Flink, Lena von Koch, Ann Charlotte Laska, Charlotte Ytterberg

**Affiliations:** ^1^ Department of Neurobiology, Care Sciences and Society Karolinska Institutet Stockholm Sweden; ^2^ Theme of Women's Health and Allied Health Professionals Karolinska University Hospital Stockholm Sweden; ^3^ Research and Development Unit for Elderly Persons (FOU nu), Region Stockholm Järfälla Sweden; ^4^ Theme of Heart and Vascular and Neuro Karolinska University Hospital Stockholm Sweden; ^5^ Department of Clinical Sciences, Danderyd Hospital Karolinska Institutet Stockholm Sweden

**Keywords:** coordination, handovers, integrated care, patient discharge, person‐centred care, rehabilitation

## Abstract

**Background:**

Care transitions from hospital to home are a critical period for patients and their families, especially after a stroke. The aim of this study was to assess the feasibility, fidelity and acceptability of a co‐designed care transition support for stroke survivors.

**Methods:**

A non‐randomised controlled feasibility study recruiting patients who had had stroke and who were to be discharged home and referred to a neurorehabilitation team in primary healthcare was conducted. Data on the feasibility of recruitment and fidelity of the intervention were collected continuously during the study with screening lists and checklists. Data on the perceived quality of care transition were collected at 1‐week post‐discharge with the Care Transition Measure. Data on participant characteristics, disease‐related data and outcomes were collected at baseline (hospitalisation), 1 week and 3 months post‐discharge. Data on the acceptability of the intervention from the perspective of healthcare professionals were collected at 3 months using the Normalisation Measure Development Questionnaire.

**Results:**

Altogether, 49 stroke survivors were included in the study: 28 in the intervention group and 21 in the control group. The recruitment and data collection of patient characteristics, disease‐related data, functioning and outcomes were feasible. The fidelity of the intervention differed in relation to the different components of the co‐designed care transition support. The intervention was acceptable from the perspective of healthcare professionals. Concerns were raised about the fidelity of the intervention. A positive direction of effects of the intervention on the perceived quality of the care transition was found.

**Conclusion:**

The study design, data collection, procedures and intervention were deemed feasible and acceptable. Modifications are needed to improve intervention fidelity by supporting healthcare professionals to apply the intervention. The feasibility study showed a positive direction of effect on perceived quality with the care transition, but a large‐scale trial is needed to determine its effectiveness.

**Patient or Public Contribution:**

Stroke survivors, significant others and healthcare professionals were involved in a co‐design process, including the joint development of the intervention's components, contextual factors to consider, participant needs and important outcomes to target.

**Trial Registration:**

ClinicalTrials.gov ID: NCT0292587.

## 1 Background

Stroke is a major global health problem, being the leading cause of death and disability worldwide [1, 2]. With its sudden onset, stroke is a stressful event for stroke survivors and their families [3], often resulting in physical and cognitive impairment and emotional burden. During the past decades, considerable improvements have been made in the prehospital and acute medical care of stroke, which has led to a decrease in the length of hospital stay [4, 5]. There is strong evidence that the acute stroke care should be supplied in hospital stroke units [6], followed by care and rehabilitation in the community setting to regain functioning. A care transition, defined as ‘a set of actions designed to ensure the coordination and continuity of healthcare as patients transfer between different locations’ [7], that is, a shift in responsibility from one healthcare setting to another, is therefore almost always necessary. There is, however, a lack of consensus on how to organise the stroke care transition [8, 9].

The transition from hospital to home is a critical period that can impose a burden on stroke survivors and significant others [10], as consequences such as physical and cognitive impairment, aphasia, poststroke fatigue and depression often contribute to stroke survivors and significant others feeling unprepared for the care transition to the home [11]. Further, a fragmented healthcare system contributes to challenges and unmet needs of stroke survivors, including accessing and navigating appropriate health services [3]. In addition, even though stroke survivors' and their families' need for information and support varies [12, 13], several studies report on their lack of information, support and skills for managing their health and recovery, feelings of uncertainty and anxiety to undertake everyday activities and adjusting to new routines [14, 15, 16, 17]. Hence, it is particularly important to address the unique situation and needs of each stroke survivor and significant other during the care transition to facilitate the rehabilitation and recovery after discharge [18, 19].

Efforts have been made to develop interventions/support to bridge the gap after discharge from the stroke unit. Evidence supports that care transitions should be addressed through a multifaceted approach and contain activities before and after discharge [20, 21, 22]. These activities include discharge and transition planning, care coordination and timely communication between care providers [23, 24]. Further education and support to develop self‐management skills among stroke survivors and their families have been suggested [25]. Although transitional support interventions have shown promise in improving functional outcomes, enhancing quality of life and reducing depression and anxiety, a recent review concluded that the essential components of an effective care transition remain unknown [26]. One reason is that care transitions are complex processes that occur within complex systems [27] and must adapt to changing individual and contextual conditions, making it difficult to pinpoint what works and for whom.

Acknowledging the complexity surrounding care transitions, we developed an intervention, a coordinated and person‐centred care transition for stroke survivors and their significant others, using the Medical Research Council framework of complex interventions [28]. The intervention was developed by identifying the evidence base for care transitions [26, 29, 30], understanding local needs and context through our pre‐studies [14, 31, 32] and conducting a co‐design process with patients, significant others and healthcare professionals [33].

Despite recognising complexity and taking a pragmatic approach with the involvement of relevant stakeholders in the design of transitional care interventions, they are still difficult to implement in a real‐world context with regard to feasibility, intervention delivery and effects [34, 35]. Hence, as highlighted in the Medical Research Council framework, it is important to investigate aspects of feasibility, acceptability and study design before conducting a large‐scale trial. Thus, the aim of this study was to assess the feasibility, fidelity and acceptability of the co‐designed care transition support for stroke survivors. The main objectives were to assess:

- the feasibility of recruitment and retention of patients and significant others,
- the fidelity of intervention,
- the acceptability of the intervention from the perspective of healthcare professionals,
- the feasibility of collecting data on participant characteristics, disease‐related data, functioning and outcomes and
- the direction of effects of the intervention on outcomes.

## 2 Methods

### 2.1 Study Design and Procedure

We conducted a feasibility study with a non‐randomised controlled design in Stockholm, Sweden, according to the procedures previously described in a study protocol [36]. The intervention was applied at one stroke unit and one geriatric ward at Hospital A, together with two corresponding neurorehabilitation teams in primary care. To assess the feasibility of the study design, a control group was recruited from a stroke unit at Hospital B and one geriatric ward at Hospital C. The screening and inclusion process was carried out from October 2021 to September 2022. In each study site, appointed health professionals conducted the screening and inclusion processes. The CONSORT 2010 statement for randomised pilot and feasibility trials [37] was used as a structure for the reporting of the study and can be seen in Appendix S1.

The ethical application was approved by the Swedish Ethical Review Authority, dnr: 2015/1923‐31/2, 2019‐04219 and 2021‐02274. The trial was registered at ClinicalTrails.gov, registration no. NCT02925871.

### 2.2 Participants

#### 2.2.1 Patients

Patients who had a first‐time or recurrent stroke and were to be discharged home from the participating stroke unit/geriatric ward and referred to a neurorehabilitation team in primary healthcare were eligible for inclusion. Exclusion criterion was inability to give informed consent, for example, due to severe aphasia. Patients were provided oral and written information about the study. All included participants gave informed consent before enrolment in the study. To evaluate the feasibility, fidelity and acceptability objectives of our study, we aimed to include 50 patients consecutively: 25 in the intervention group and 25 in the control group [38].

#### 2.2.2 Significant Others

Included patients were asked to name a significant other for potential participation in the study. If patients provided a name and contact details, written information about the study, a form for informed consent and a postage‐paid, self‐addressed return envelope were sent to the significant others. Significant others who returned a signed informed consent were included.

#### 2.2.3 Healthcare Professionals

Physicians and allied health professionals (physiotherapists, occupational therapists and speech and language therapists) from the stroke unit and the geriatric ward at Hospital A and the two corresponding neurorehabilitation teams were invited to participate. The healthcare professionals were provided oral and written information about the study. Included participants gave written informed consent before enrolment.

#### 2.2.4 Supported Care Transition—Intervention

The intervention was based on our pre‐studies [14, 31, 32] and the stroke care trajectory literature [6, 29, 30], and was developed in a co‐design process using design thinking methodology involving stroke survivors, significant others and healthcare professionals from Hospitals A and B and corresponding neurorehabilitation teams in primary care [33]. The intervention aimed at patients', significant others' and healthcare professionals' shared understanding of the patient's needs and preferences; patient preparedness for homecoming; and coordination within and between the stroke units at the hospital and the neurorehabilitation teams in primary care. The components were intended to be used based on individual needs and included different modes of information related to the care and rehabilitation after stroke, an individualised discharge letter, a ‘what matters to me’ dialogue, a bridging e‐meeting, the Teach Back method [39, 40] and a safe instant messaging system. Details of the intervention components are presented in Table 1. The intervention was delivered by physicians and allied health professionals at the stroke unit and geriatric ward and allied health professionals in the two corresponding neurorehabilitation teams.

**Table 1:** Overview of the intervention components.

Intervention components	Content of intervention component
Modes of information	Three different modes of information about the next rehabilitation phase were offered to patients and significant others and presented by the allied health professionals at the hospital: (1) general informational video (3 min long), including general information about the neurorehabilitation teams in primary care and the rehabilitation process; (2) specific informational video (3 min long) containing a presentation of and by the neurorehabilitation team (physiotherapist, occupational therapist, speech and language therapist and social worker) from the specific neurorehabilitation team responsible for the continued rehabilitation after discharge and (3) an informational pamphlet, size A5, four pages, was provided to patients before discharge by the allied health professionals at the hospital, including a general introduction to the neurorehabilitation team and information about the subsequent rehabilitation in the home environment. The pamphlet also contained a QR code and link to the informational video and contact information for the specific neurorehabilitation team responsible for continued rehabilitation after discharge from the stroke unit.
Individualised discharge letter	Physicians at the hospital provided an individualised discharge letter to ensure that the discharge information would include information about the diagnosis, results of examinations, recommended lifestyle changes, car driving, follow‐ups, what to consider after discharge and where to turn with questions. Further, the medication list was reviewed to ensure that patients received appropriate medication and dosages. This information was shared with patients before discharge to help them understand their medications better, including their purpose, how to take them and any potential side effects.
What matters to me dialogue	A dialogue about ‘what matters to me’ focusing on the patient's preferences, thoughts and wishes regarding the care and rehabilitation at the stroke unit, as well as concerns about coming home and the continued rehabilitation in the home environment. This dialogue was organised by the allied health professionals at the hospital and was intended to be continued after discharge together with the neurorehabilitation team.
Bridging e‐meeting	‘A bridging e‐meeting was offered before discharge to coordinate the discharge and care transition. The e‐meeting was arranged by allied health professionals at the hospital and coordinated with the neurorehabilitation team. The purpose was to connect the patient, their significant other, and allied health professionals at the hospital with the neurorehabilitation team responsible for subsequent rehabilitation after discharge. The e‐meeting consisted of a personal introduction; a discussion about the patient's situation, needs, wishes and concerns about returning home; a presentation from the home rehabilitation team about their services; an opportunity for the patient and significant other to ask questions and an agreement on an appointment for the first home visit’.
The Teach Back method	The Teach Back method [37, 38] was used to facilitate dialogue and a shared understanding of the patient's situation and the healthcare professionals' information. Teach Back is an iterative communication method that verifies patient understanding by asking the patient to repeat or summarise what was said during the conversation. The method allows people involved in the conversation to highlight potential differences in what is perceived from a conversation. Teach Back was intended to be used in the conversations around the information videos and pamphlets, during ‘what matters to me dialogue’, the bridging e‐meeting and discharge encounters.
Safe instant messaging system	A safe instant messaging system within the electronic health record between the staff at the hospital and the neurorehabilitation team in primary care was made available. The intention was to facilitate inter‐organisational communication to ensure the exchange of information and resolve remaining questions regarding patient care and rehabilitation.

#### 2.2.5 Healthcare Professional Training

Physicians and allied health professionals (physiotherapists, occupational therapists and speech and language therapists) from the stroke unit and the geriatric ward at Hospital A and the two corresponding neurorehabilitation teams (physiotherapists, occupational therapists, speech and language therapists and social workers) were offered training in the components of the intervention. The education consisted of one educational video about the intervention, with focus on the intention and practical application of Teach Back, followed by practice and reflections about the performance. Role play and clinical scenarios with audit and feedback were offered. All participants were encouraged to practice and reflect. As the physicians at the hospital altered during the study period, continuous education on Teach Back was offered. Informational ‘pocket reminders’ about the intervention components were provided to all physicians and allied health professionals.

#### 2.2.6 Standard Care Transition—Control

Control groups at Hospitals B and C received standard care transitions. Based on our pre‐studies [14, 32], ‘standard care transitions’ entailed a discharge meeting with the patient and responsible physicians and a standard discharge letter about the hospitalisation. The care transition was initiated by an electronic referral from allied health professionals at the hospital to the receiving neurorehabilitation team. The referral notified the neurorehabilitation team about the patient's discharge. The teams were then obliged to contact the patient within 48 h after hospital discharge.

### 2.3 Data Collection

Data on feasibility of recruitment, retention and fidelity of the intervention were collected continuously during the study with screening lists and checklists. Data regarding patient characteristics, disease‐related data, functioning and outcomes were collected at baseline (hospitalisation), 1 week and 3 months post‐discharge. Data on significant other characteristics and outcomes were collected at 3 months. Data concerning the acceptability of the intervention were collected at 3 months using questionnaires. Data were collected by the research team.

#### 2.3.1 The Feasibility of Recruitment and Retention of Patients and Significant Others

We defined recruitment rate as the number of patients included from the eligible patients, which was recorded on the subject screening list and the subject enrolment list. Attrition and reason for drop‐out were registered in conjunction with the data collection time points.

#### 2.3.2 The Fidelity of Intervention

Fidelity of the intervention was defined as the extent to which the intervention was delivered as planned. Fidelity was assessed by the frequency of participants in the intervention group receiving the different components of the intervention. The data were collected by the first author using a checklist for each patient. The checklist included the different intervention components that were assessed as delivered or not delivered for each included study participant (stroke survivor).

#### 2.3.3 The Acceptability of the Intervention From the Perspective of Healthcare Professionals

Data on the acceptability of the intervention were collected using the Normalization MeAsure Development Questionnaire (NoMAD) [41], based on the Normalization Process Theory [42]. NoMAD was measured 3 months into the study and consists of 23 items. The first three questions assess familiarity and normality on a scale from 1 (*not at all*) to 10 (*completely*) and were categorised into as ‘completely new/no’ (1–4), ‘partly’ (5) and ‘familiar/yes’ (6–10). The remaining 20 questions are divided into four constructs: coherence/sense‐making (four items), cognitive participation (four items), collective action (seven items) and reflexive monitoring (five items) [42], scored on a 5‐point Likert Scale from 1 (*strongly disagree*) to 5 (*strongly agree*). A mean score is calculated per item.

#### 2.3.4 The Feasibility of Collecting Data on Participant Characteristics, Disease‐Related Data, Functioning and Outcomes

Completeness of data collection and outcome measures were registered and described as the number and percentages of collected measures related to the different time points of data collection. In the procedure for collecting patient data, the questionnaires were mailed to the patient's home addresses with information that a research assistant would call the patient to collect data in a structured interview.

### 2.4 Patients

#### 2.4.1 Characteristics, Disease‐Related Data and Functioning

Data on participant characteristics, disease‐related data and functioning were collected at baseline, 1 week and 3 months after discharge from hospital. Baseline data were collected before the hospital discharge on participant characteristics, comprising age, sex, educational level (elementary/secondary or university/college), civil status (living alone or cohabiting), work status (working or not working), economic status (insufficient, just enough, good enough), information on the use of home care services before and after stroke (yes or no), using a questionnaire.

Participants' disease‐related data comprising length of stay, type of stroke (ischaemic stroke or intracerebral haemorrhage), stroke severity assessed using the National Institute of Health Stroke Scale (NIHSS) [43], reperfusion therapy (yes or no), aphasia, comorbidity and ability to perform activities of daily living (ADL) were retrieved from patients' medical records. The Charlson Comorbidity Index [44] was used to classify comorbidity into 3 levels of severity: no comorbidity (scores 0), low (scores 1 and 2) and moderate or severe (scores > 2).

The Barthel Index (BI) was used to assess ADL [45]. The BI assesses independence in 10 personal care and mobility activities. Scores range from 0 to 100, where a higher score reflects a higher degree of independence. The modified Rankin Scale (mRS) was used to assess the degree of disability [46]. The mRS scores range from 0 (*no disability*) to 6 (*death*) and were categorised as mild (0–1), moderate (2–3) and severe (4–6) disability [47]. A short version of the Montreal Cognitive Assessment Scale (Mini‐MoCA) was used to assess cognitive function [48, 49]. Scores range from 1 to 15, and scores < 11 indicate cognitive impairment. The Patient Health Questionnaire‐2 was used to assess depressive symptoms [50]. Score ranges from 0 to 6, and a score of > 3 is considered to indicate depressive symptoms. Walking ability was categorised as walks independently without aid and support, walks with walking aid or walks with assistance and support/unable to walk. Perceived recovery after stroke was rated by the participants on the subscale of the Stroke Impact Scale 3.0, a visual analogue scale from 0 (*not recovered at all*) to 100 (*fully recovere*d) [51, 52]. Perceived fatigue after stroke was rated by the participants on a visual analogue scale from 0 (*no fatigue*) to 100 (*extreme fatigue*).

#### 2.4.2 Outcomes

Data on perceived quality of care transitions were collected using the Swedish version of the Care Transition Measure (CTM‐15) 1 week after discharge from hospital [53, 54, 55]. The CTM‐15 is a unidimensional scale assessing the overall perceived quality in four areas of importance for quality care transitions. The 15 items are rated on a 4‐point Likert scale ranging from 1 (*strongly disagree*) to 4 (*strongly agree*) [56]. For each item, an additional response of ‘don't know/not applicable’ is available. The items are summarised and computed in a linear transformation into a total score of 0–100 [57]. The total score reflects the overall perceived quality of the care transition, with lower scores indicating a poor‐quality care transition and higher scores indicating a higher quality care transition.

Other outcomes were collected at 1 week and 3 months. Health literacy was assessed using the Health Literacy Questionnaire (HLQ) [58]. The HLQ comprises nine scales that each measures an aspect of health literacy. The participants are asked to answer each question on a Likert scale ranging from 1 (*strongly disagree or cannot do*) to 5 (*strongly agree or very easy*). The mean score is calculated for each subscale.

Adherence to medical treatment was assessed using the Swedish version of the Medication Adherence Report Scale (MARS‐5) [59]. The MARS‐5 is a self‐report scale containing five items assessing nonadherent behaviour. The respondent is asked to agree or disagree with each statement on a Likert scale ranging from 1 (*always*) to 5 (*never*). The total score ranges from 5 to 25, where higher scores indicate higher adherence.

Knowledge of information about medical treatment was assessed using open‐ended questions concerning knowledge of which new medications had been prescribed at the time of discharge from hospital and/or there had been any changes in previous medications, as well as knowledge of the reasons for new medications and/or changes in medication. Responses were verified against medical records and dichotomised into has knowledge or has no knowledge.

The General Self‐Efficacy Scale (GSES) was used at 3 months to assess general perceived self‐efficacy [60]. The questionnaire consists of 10 items and responses are made on a 4‐point Likert scale. The score ranges between 10 and 40, where higher scores indicate higher perceived self‐efficacy.

### 2.5 Significant Others

#### 2.5.1 Characteristics

Data on significant other characteristics comprising age, sex, educational level (elementary/secondary or university/college), civil status (living alone or cohabiting), work status (working or not working) and economic status (insufficient, just enough, good enough) were collected using questionnaires.

Information about whether the significant others provided support to the stroke survivor before and/or after the hospital stay, in personal ADL (P‐ADL) and instrumental ADL (I‐ADL) or with other matters was collected with multiple‐choice questions graded on a 3‐point scale (yes, partly, no).

#### 2.5.2 Outcomes

The Caregiver Burden Scale (CBS) was used to assess the subjective burden on significant others [61]. The CBS consists of 22 items scored on a 4‐point Likert scale (1–4). The total score ranges from 22 to 88 points, with higher scores indicating a higher burden on the significant other. The EQ VAS was used to assess significant others' self‐rated health on a visual analogue scale, ranging from 0 ‘The worst health you can imagine’ to 100 ‘The best health you can imagine’. Life satisfaction in significant others was assessed with the global satisfaction with life item from the Life Satisfaction checklist [62, 63]. The item is scored on a Likert‐scale from 1 (*very dissatisfying*) to 6 (*very satisfying*) and was categorised into ‘satisfied’ (≥5) or ‘not satisfied’ (≤4).

### 2.6 Analyses

Descriptive statistics (e.g., mean and standard deviation, median and interquartile range, 95% confidence interval) were used to present the measures of feasibility and participants' characteristics, disease‐related data and functioning. To analyse differences between participants who received the intervention and those who did not, the Mann–Whitney *U* test or Student's *t* test was used for continuous data and Fisher's Exact Test was used for categorical data. The level of significance was set at *p* ≤ 0.05.

## 3 Results

### 3.1 The Feasibility of Recruitment and Retention of Patients and Significant Others

**Figure 1:**
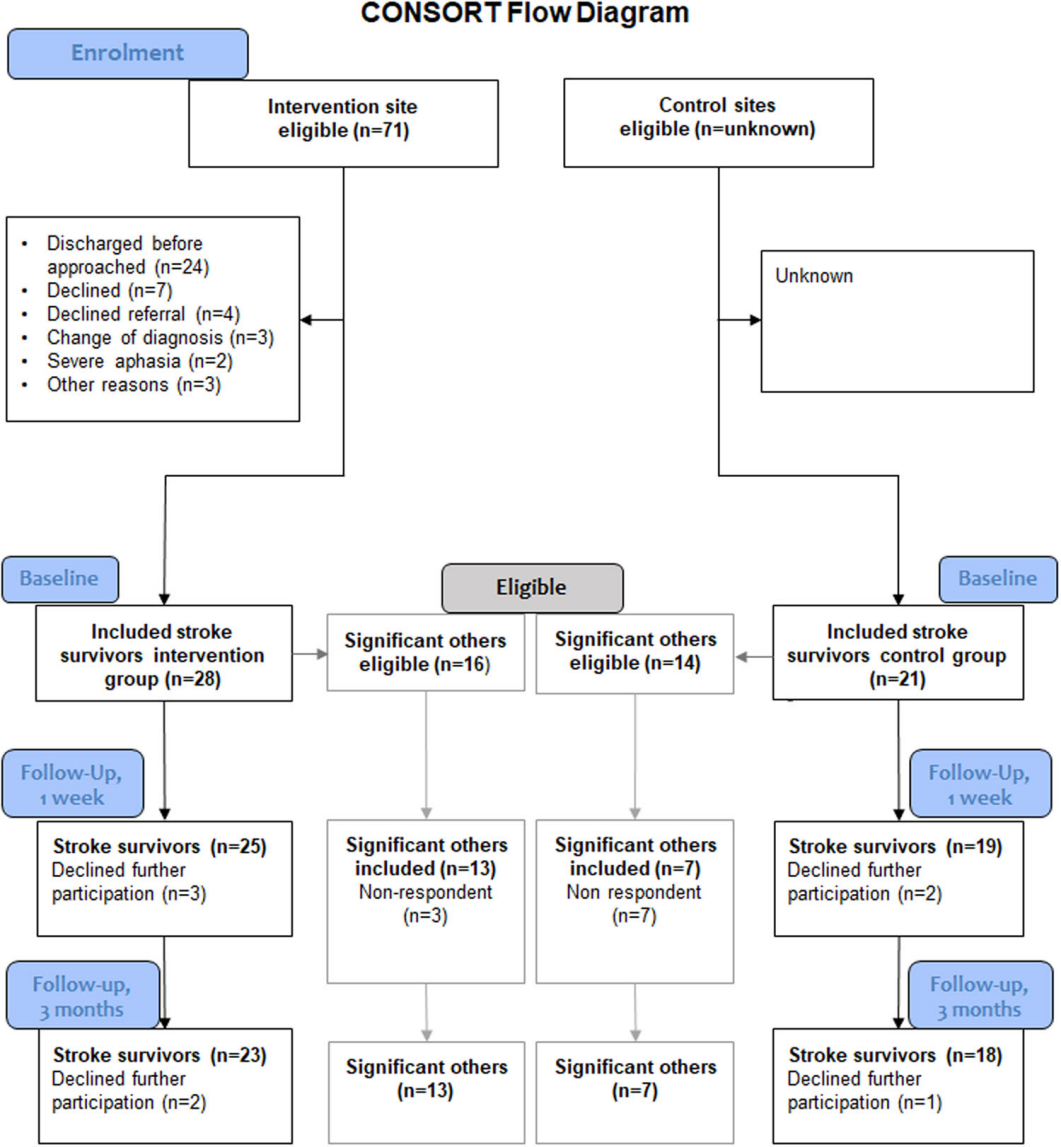
Flowchart of included participants and time point for follow‐up.

At the intervention site (Hospital A), 71 patients were eligible for inclusion in the study during the study period; of these, 28 participants were included, a recruitment rate of 39%. Figure 1 illustrates the flowchart of the recruitment of patients and significant others. In the control site (Hospital B), 21 participants were included. The screening protocol only included the 21 participants included in the study and no participants were recruited from Hospital C. No information was obtained on number of eligible patients at the control sites; hence, the recruitment rates are unknown.

The reasons for not being included in the study were patients being discharged before invitation to participate, declining participation, declining referral to continued rehabilitation with neurorehabilitation team in primary care, change of diagnosis, severe aphasia or other reasons such as not feeling recovered enough to participate. The intervention group was recruited from October 2021 to December 2021, a recruitment rate of nine participants per month. The control group was recruited from February 2022 to September 2022, with a recruitment pace of three patients per month. Eight patients declined further participation during the study period, five from the intervention group and three from the control group, with a total drop‐out rate of 16%. The reasons for drop‐out are presented in Figure 1.

Altogether, 49 stroke survivors and 20 significant others were included in the study. Demographics and clinical characteristics of the participants are presented in Table 2. The mean age of the stroke survivors was 75 years; 55% were men, 37% were living alone, 18% had a moderate/severe disability and 12% had aphasia. The baseline demographics and characteristics were comparable, except for level of education and length of stay. In the intervention group, 46% had a university degree in comparison to 19% in the control group (*p* = 0.027). The mean length of stay in the intervention group was 4 days in comparison to the control group, who had a mean length of stay of 2 days (*p* = 0.007).

**Table 2:** Demographics and clinical characteristics of the stroke survivors.

Variable	Total, *n* = 49	Intervention, *n* = 28	Control, *n* = 21	*p* value
Patients				
Age, median (IQR) min–max	75 (69–82), 27–94	77 (72–85), 27–94	72 (66–78), 48–90	0.077^a^
Sex, male, *n* (%)	27 (55)	15 (54)	12 (57)	1.0^b^
Education, *n* (%)^c^				0.029^b^
Elementary/secondary	25 (51)	10 (36)	15 (72)	
University	17 (35)	13 (46)	4 (19)	
Economy, *n* (%)^c^				0.530^b^
Insufficient	1(2)	0 (0)	1 (5)	
Just enough	18 (37)	11 (39)	7 (33)	
Good enough	24 (49)	13 (45)	11 (52)	
Cohabiting, *n* (%)	31 (63)	15 (64)	16 (76)	0.139^b^
Working, *n* (%)	11 (22)	7 (25)	4 (19)	0.728^b^
Help from home care services before stroke, *n* (%)	3 (6)	2 (7)	1 (5)	0.322^b^
Help from home care after stroke, *n* (%)	3 (6)	2 (7)	1 (5)	0.331^b^
Length of stay, days, median (IQR), min–max	3 (2–5), 1–15	4 (2–8), 1–15	2 (2–3), 1–6	0.007^a^
Type of stroke, *n* (%)				0.569^b^
Ischaemic	46 (94)	27 (96)	19 (91)	
Intracerebral haemorrhage	3 (6)	1 (4)	2 (9)	
Reperfusion therapy, *n* (%)	4 (8)	2 (7)	2 (9)	
NIHSS, median (IQR) min–max	2 (1–3), 0–18	2 (1–3), 0–18	2 (1–3), 0–13	0.773^a^
Aphasia, *n* (%)	6 (12)	3 (10)	3 (14)	0.795^b^
Comorbidity, *n* (%)				0.756^b^
No comorbidity	23 (47)	12 (43)	11 (52)	
Low comorbidity	22 (45)	13 (46)	9 (43)	
Moderate/severe comorbidity	4 (8)	3 (11)	1 (5)	
Disability, *n* (%)				0.229^b^
Mild	31 (63)	15 (54)	16 (76)	
Moderate	17 (35)	12 (43)	5 (24)	
Severe	1 (2)	1 (3)	0 (0)	
Cognitive impairment, *n* (%)	18 (37)	9 (32)	9 (43)	0.763^b^
Depressive symptoms, *n* (%)	11 (22)	5 (18)	6 (29)	
Barthel Index, median (IQR), min–max	100 (100–100), 25–100	100 (100), 25–100	100 (100), 90–100	0.175^a^
Walking ability, *n* (%)				1.0^b^
Walks independently without aid and support	37 (76)	20 (72)	17 (81)	
Walks with walking aid	10 (20)	6 (21)	4 (19)	
Walks with assistance and support/unable to walk	2 (4)	2 (7)	0 (0)	
Self‐rated recovery, median (IQR), min–max	73 (60–90), 20–100	75 (60–90), 20–100	65 (55–90), 25–99	0.418^a^
Fatigue, median (IQR), min–max	50 (20–70), 0–99	50 (13–69), 0–90	50 (30–80), 5–99	0.197^a^

Abbreviations: IQR, interquartile range; NIHSS, National Institute of Health Stroke Scale.

^a^ Mann–Whitney *U* test.

^b^ Fisher exact test.

^c^ Missing: *n* = 6.

Out of the included stroke survivors, 30 had eligible significant others (16 in the intervention group, 14 in the control group). A total of 20 significant others were included (13 in the intervention group, 7 in the control group), resulting in a 64% recruitment rate. The demographics and characteristics of the significant others can be seen in Appendix S2. The significant others' mean age was 74 years, with 30% men, 35% not living together with the stroke survivor and 30% working. Additionally, 10% assisted in P‐ADL and 55% in I‐ADL. The demographics and characteristics of the significant others were comparable between the intervention and control groups.

### 3.2 The Fidelity of the Intervention

All stroke survivors, except four, received the pamphlet (86%), and half of the stroke survivors received the generic informational video (50%). A ‘what matters to me’ dialogue was conducted with 17 (61%) stroke survivors. Allied health professionals used the Teach Back method with 15 (54%) of the stroke survivors and physicians with 19 (68%) of the stroke survivors during the discharge encounters. Details on the fidelity of the intervention are presented in Table 3.

**Table 3:** Frequency of stroke survivors in the intervention group receiving the intervention.

Intervention component	Yes, *n* (%)
Generic video	14 (50)
Specific video	7 (25)
Pamphlet	24 (86)
Specific contact details	8 (29)
What matters to me dialogue	17 (61)
Teach Back—allied health professionals	15 (54)
Teach Back—physician	19 (68)
Bridging e‐meeting	1 (4)
Individualised discharge letter	21 (75)
Review of medication list	19 (68)

#### 3.2.1 The Acceptability of the Intervention From the Perspective of Healthcare Professionals

In total, 21 healthcare professionals from the intervention sites answered the NoMAD, including 14 physicians and 7 allied health professionals; for background information, see Table 4.

**Table 4:** Background information on healthcare professionals answering the NoMAD questionnaire.

Variable	Value
Sex, female, *n* (%)	14 (67)
Age, mean (SD) min–max	37 (11), 26–65
Setting	
Stroke unit	17 (81%)
Geriatric ward	4 (19%)
Profession, *n* (%)	
Physician	14 (66)
Occupational therapist	4 (19)
Speech and language therapist	1 (5)
Physiotherapist	2 (10)
Years within profession, mean (SD) min–max	7.5 (10), 1–42
Years at current workplace, *n* (%), years	
< 1	6 (29)
1–2	8 (38)
3–5	3 (14)
6–10	0
11–15	1 (5)
> 15	3 (14)

**Figure 2:**
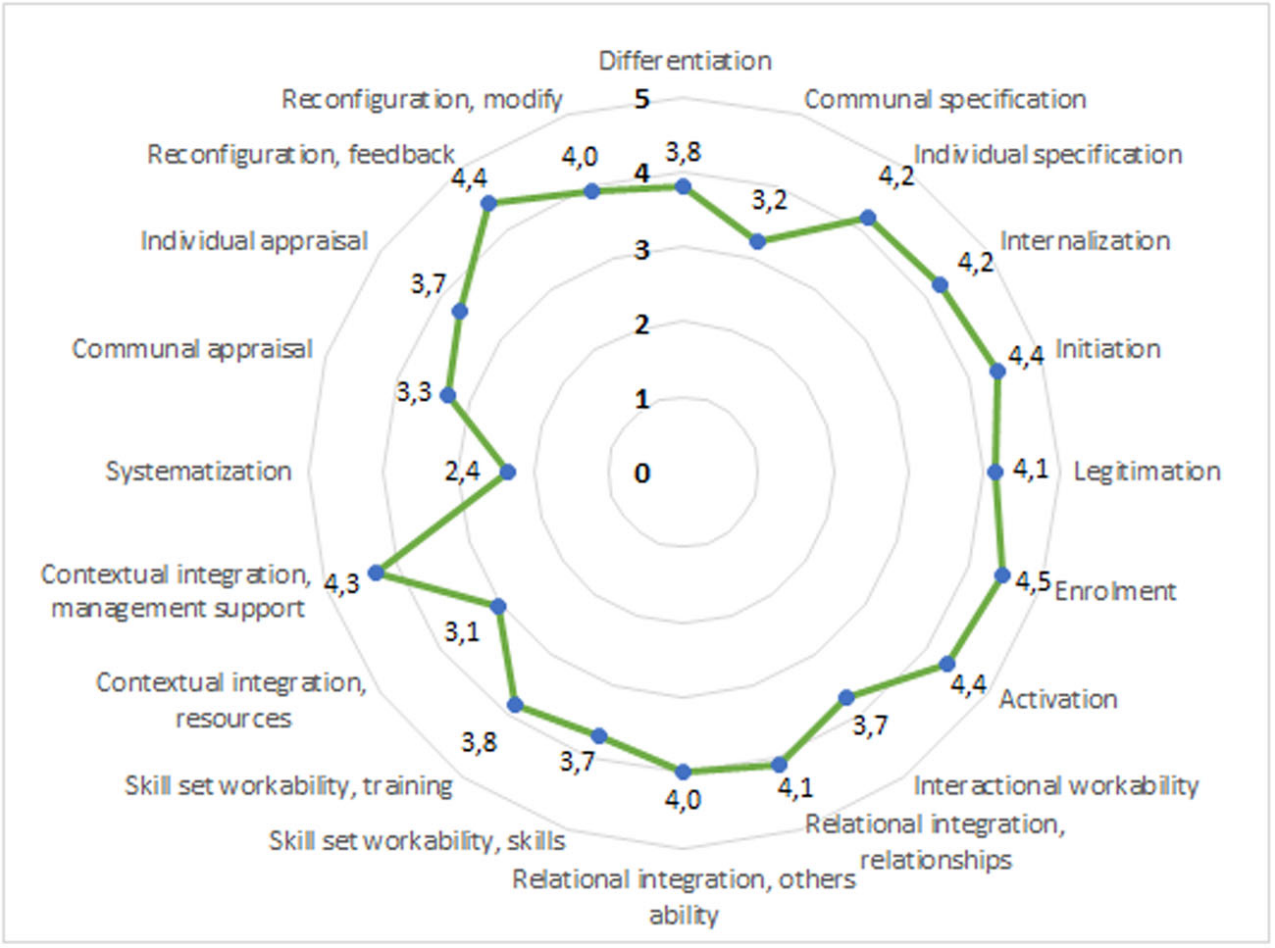
Petal chart showing the average scores for the 20 items of NoMAD on a Likert scale of 1 (*strongly disagree*) to 5 (*strongly agree*), *n* = 21.

A majority of the healthcare professionals (62%) reported that the intervention was familiar when performing tasks related to the intervention; 57% reported that work related to the intervention currently was a natural part of their work and 81% reported that the work related to the intervention could become a natural part of their work. The scoring on each of the 16 items of the NoMAD is presented in Figure 2. One item, systematisation (I am aware of reports about the effects of the intervention), scored below average, with a mean score 2.4. In Appendix S3, the exact wording of each item is presented.

#### 3.2.2 The Feasibility of Data Collection on Participant Characteristics, Disease‐Related Data, Functioning and Outcomes

At baseline, there was 99% completeness of the collected data. The MoCA was not collected from one participant due to language barriers. The CTM‐15 had 100% completeness at 1 week. For other outcomes at 1 week, one of the participants did complete the HLQ due to fatigue, rendering 98% data completeness. At 3 months, one questionnaire was lost in the mail; three participants could not complete the MoCA and one participant could not complete all questionnaires due to fatigue. Data from significant others resulted in 100% completeness.

#### 3.2.3 The Direction of Effects of the Intervention on Outcomes

**Figure 3:**
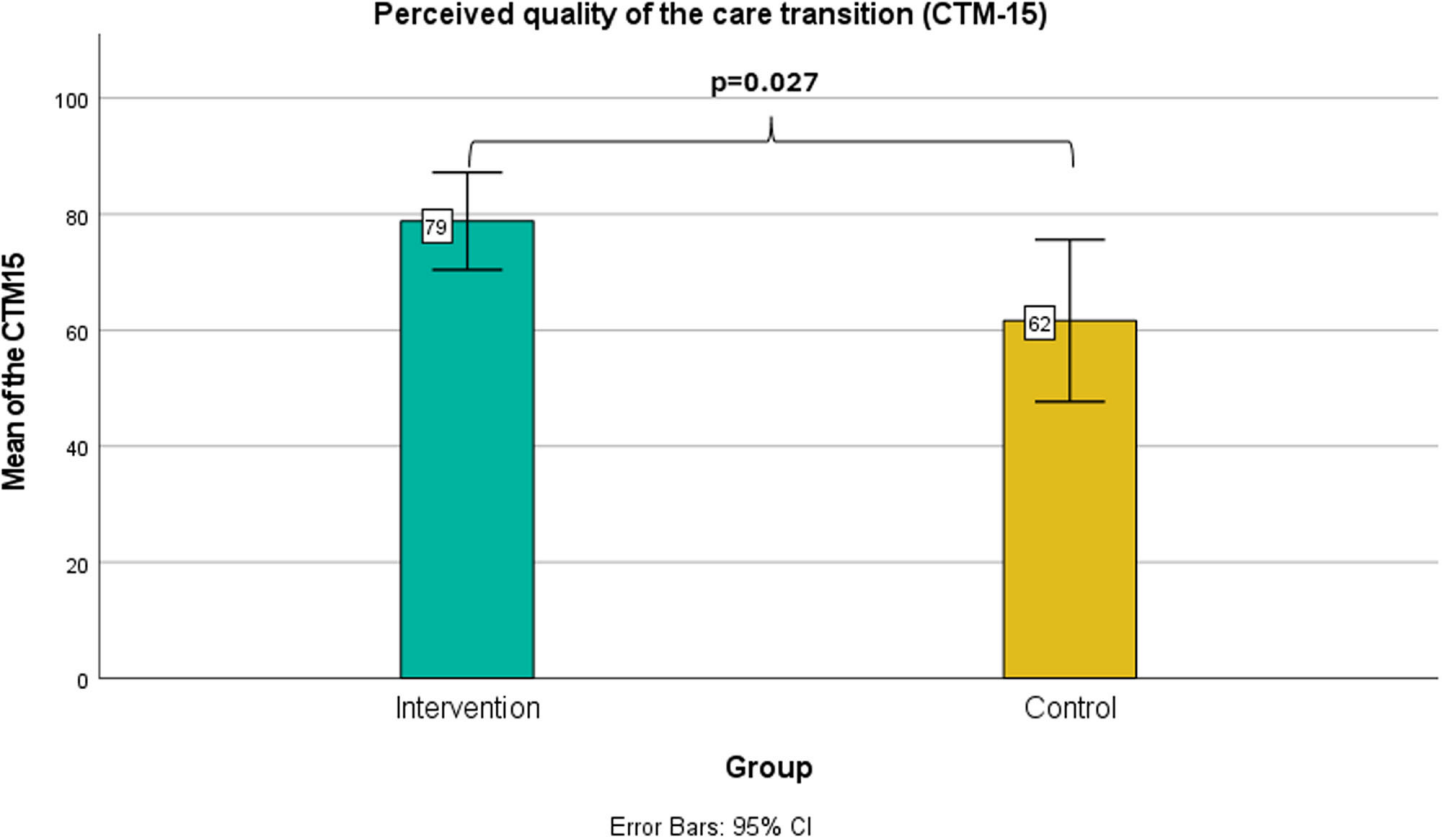
Error bar chart of the perceived quality of the care transition between the intervention and control groups.

A significant difference between groups was found regarding the perceived quality of the care transition, with the intervention group reporting a higher perceived quality (mean = 79) compared to the control group (mean = 62, *p *= 0.027). Error bar charts illustrating the variability of data and mean values are shown in Figure 3. Other outcomes at 1 week and 3 months, detailed in Appendices S4 and S5, showed no significant differences in health literacy, medication adherence and knowledge or functioning. Additionally, no significant differences were found between groups for outcomes of significant others, as shown in Appendix S2.

## 4 Discussion

The aim of this study was to assess the feasibility, fidelity and acceptability of the co‐designed care transition support for stroke survivors to inform a large‐scale trial. The recruitment and data collection of patient characteristics, disease‐related data, functioning and outcomes were found to be feasible. A positive direction of effects of the co‐designed care transition support on the outcome perceived quality was found. The intervention was acceptable from the perspective of healthcare professionals. The fidelity of the intervention indicated a need to address strategies to improve the implementation of the intervention's components before entering a large‐scale trial.

The recruitment rate at the intervention site was higher than that in similar stroke trials [64, 65]. Despite this, there is room for improvement, as a low recruitment rate can lead to selection bias and threats to the external validity and generalisability of the study findings. During the 3‐month study period, 24 eligible patients were discharged before being asked to participate due to the short length of stay at the acute stroke unit and a narrow time frame for decisions on discharge. Hence, we sometimes missed the opportunity to ask patients to participate. As the assigned healthcare professionals responsible for the screening and inclusion could not always be present, we sometimes missed the opportunity to invite patients to participate in the study. The limited opportunity for people responsible for screening and inclusion to be present on site is a well‐known barrier concerning stroke trials [66], related to factors such as lack of resources and weak infrastructure for research in the clinical environment [67].

Despite having the same recruitment and data collection routines at intervention and control sites, we ended up with an unknown number of eligible patients and slower recruitment in the control sites. Having healthcare professionals conduct the recruitment and data collection on their own might be sensitive to factors such as workload and lack of time and resources devoted to research [68]. This is something to consider when planning for a larger trial, either by having a more continuous follow‐up with education in good clinical practice or having a dedicated study coordinator at the control sites. These strategies have previously been used to improve recruitment in research [69].

The fidelity of the intervention varied among the different components of the intervention. The variation was, to some degree, expected as the components of the intervention were intended to be used based on individual needs,; hence, all components might not have been deemed necessary for each patient. Nevertheless, we had expected a higher fidelity rate across the intervention components. The variation in fidelity could be explained by how the components are understood and operationalised. We found that handing out a pamphlet or the individualised discharge letter was done to a greater extent than conducting the ‘what matters to me dialogue’ or using the Teach Back methodology. Fidelity is influenced by the degree of complexity of intervention components [70]. Using components that are more familiar and closer to everyday clinical practice is more accessible than using components that challenge traditional practice and structures [71]. Further, as interventions that depend on a greater proportion of behaviour change in healthcare professionals also need to be followed by strategies focusing on collective action and reflexive monitoring, a future large‐scale trial needs to consider how to best support action and education as part of incorporating the intervention in clinical practice [72]. In addition, considerations need to be made regarding how to best assess fidelity, given that the intervention is flexible in the sense that it is delivered based on the needs of the individual. Before conducting a large‐scale trial, it is also important to evaluate the benefit and value of each intervention component from the perspective of stroke survivors and healthcare professionals to avoid unnecessary complexity and workload.

The ‘what matters to me dialogue’ was conducted with two‐thirds of the patients and the Teach Back method was used with half of the patients by allied health professionals and two‐thirds by physicians. The results indicate that the use of these methods was feasible. However, the fidelity was not considered satisfactory. More knowledge is warranted on why some patients did not receive these parts of the intervention, which is currently being explored in a parallel process evaluation. Lack of skills or confidence, organisational support and time constraints have been described as barriers when implementing person‐centred communication [71, 73]. In a previous study within the same context, we found that time pressure and administrative duties were barriers to why communication with patients was overlooked [14]. Even though training was offered to all healthcare professionals, it was not mandatory, which might have affected the results. A recent review reported that the education and support of clinicians in using Teach Back should be part of the implementation strategy [74]. A future large‐scale trial should consider how to best support healthcare professionals in developing communication skills and integrating person‐centred communication as a natural part of clinical practice.

Only one bridging e‐meeting was conducted, even though cross‐organisational communication was identified as a potential solution to improve the care transition in our pre‐studies [31, 33]. One explanation could be that the bridging e‐meeting could only be conducted when patients were referred to one of the two participating neurorehabilitation teams. In addition, as the study progressed, it became evident that allied health professionals at the stroke unit did not have time to plan and conduct the e‐meeting due to the short length of stay. Hence, the selection of participants was limited, which is one explanation for the low fidelity. The use of digital support to increase coordination across organisational boundaries has shown promising results [75, 76]. However, integrating digital technologies in clinical practice has been described as challenging due to difficulties related to the fit with existing systems and clinical workflow and the risk of increased workload for healthcare professionals [77, 78].

The intervention was considered acceptable from the perspective of the healthcare professionals, which shows the potential of the intervention to be implemented in everyday clinical practice. However, the NoMAD also showed areas in need of improvement. Awareness of the intervention effects was low. This is an important finding, as knowledge about the effect of the intervention is vital in terms of the appraisal work that people do to assess and understand how a new set of practices affects them and others around them [42]. Other low scores related to a shared understanding of the purpose of the intervention and that sufficient resources were available to support the intervention. A future large‐scale trial should consider incorporation of an implementation plan, team involvement, comprehensive education and involving leadership to support the intervention, as recommended to facilitate implementation of a complex intervention in the stroke care trajectory [79].

The co‐designed care transition support showed a positive direction of effects on the perceived quality of the care transition in favour of the intervention group. This aligns with previous studies exploring multicomponent interventions targeting care transitions, which have been seen to influence the perceived quality of the care transition [80, 81], patient safety and satisfaction [82] in populations other than stroke. However, due to the small study sample, a large‐scale trial is needed to determine its effect.

The intervention and control groups were similar regarding baseline values, except for education level and length of stay, which must be considered when interpreting the results. The difference in length of stay can be explained by the fact that the control group did not include any patients from the geriatric ward who had a longer length of hospital stay [32]. For a larger trial, there is a need to consider a matched sample approach by using comparable recruitment sites regarding socioeconomic standards and sites with patients from the geriatric ward. Another limitation was the lack of knowledge about eligible patients at the control sites. This might be because we did not have a study coordinator responsible for recruitment at the control sites due to limited resources. A future large‐scale trial needs to consider changes to the recruitment process to avoid this fault. Another limitation was the use of a checklist, with partly self‐reported data to assess the fidelity of the intervention, which might lead to bias. A future trial should consider using a multi‐faceted approach to strengthen the reliability of the fidelity assessment. We had no mandatory education or training in the intervention's different components to not impose any extra burden on healthcare professionals. Further, we only invited physicians and allied health professionals to participate in the intervention, which might have affected the results. In addition to the results presented in this study, we will present results based on data collected through observations and interviews in a future process evaluation study. This study and the process evaluation will inform the development of the intervention and a future large‐scale trial.

## 5 Conclusions

Our findings indicate that recruitment and data collection were feasible. However, slower recruitment rates were noted at control sites. Robust recruitment strategies and dedicated healthcare professionals are necessary to ensure consistent participant inclusion across sites in a future full‐scale trial. Healthcare professionals found the intervention acceptable, and a positive trend was observed concerning the perceived quality of care transitions favouring the intervention group. The fidelity of the intervention components varied, with simpler components showing higher utilisation than more complex components. This variability suggests the need for enhanced strategies to support the consistent implementation of all intervention components. The co‐designed care transition support shows promise, particularly in improving the perceived quality of care transitions. However, a large‐scale trial is necessary to determine its effectiveness.

## Author Contributions

**Sebastian Lindblom:** conceptualisation, investigation, writing–original draft, methodology, validation, visualisation, writing–review and editing, formal analysis, project administration, and data curation. **Maria Flink:** conceptualisation, investigation, writing–original draft, methodology, validation, writing–review and editing, project administration, and supervision. **Lena von Koch:** conceptualisation, investigation, validation, writing–review and editing, project administration, funding acquisition, methodology, supervision, and resources. **Ann Charlotte Laska:** conceptualisation, investigation, methodology, validation, visualisation, writing–review and editing, project administration, supervision, and resources. **Charlotte Ytterberg:** conceptualisation, investigation, funding acquisition, methodology, validation, visualisation, writing–review and editing, project administration, supervision, and resources.

## Ethics Statement

The study was conducted in accordance with the Declaration of Helsinki and approved by the Swedish Ethical Review Authority dnr: 2015/1923‐31/2, 2019‐04219 and 2021‐02274.

## Consent

Informed consent was obtained from all subjects involved in the study.

## Conflicts of Interest

The authors declare no conflicts of interest.

## Supporting information

Supporting information.

Supporting information.

Supporting information.

Supporting information.

Supporting information.

## Data Availability

The data sets generated and/or analysed during the current study are not publicly available but can be available upon reasonable request. As data can indirectly be traced back to the study participants, according to the Swedish and EU personal data‐sharing legislation, access can only be granted upon request. Request for access to the data can be put to our Research Data Office (rdo@ki.se) at Karolinska Institutet and will be handled according to the relevant legislation. In most cases, this will require a data processing agreement or similar with the recipient of the data.
