# Supplementary material for: Feasibility, Fidelity and Acceptability of a Person‐Centred Care Transition Support Intervention for Stroke Survivors: A Non‐Randomised Controlled Study
Source: Health Expect. 2024 Oct 7;27(5):e70057. doi: 10.1111/hex.70057 (PMC11456962; doi:10.1111/hex.70057)
Supplement: Supplementary file 2 — Supporting information. [file HEX-27-e70057-s002.docx]

**Appendix 2**. Demographics, characteristics, and outcomes of the significant others.

| **Variable** | **Total, n=20** | **Intervention, n=13** | **Control, n=7** | **p-value** |
| --- | --- | --- | --- | --- |
| **Demographics, characteristics** |  |  |  |  |
| Age, median (IQR) min-max | 74 (63-80) 27-90 | 73 (64-80) 27-90 | 76 (61-80) 59-83 | 0.781^a^ |
| Sex, male, n (%) | 6 (30) | 3 (23) | 3 (43) | 0.613^b^ |
| Relation, n (%) |  |  |  | 0.139 ^b^ |
| Husband/wife | 9 (45) | 4 (31) | 5 (71) |  |
| Domestic partner | 3 (15) | 2 (15) | 1 (14) |  |
| Living apart | 3 (15) | 3 (23) | 1 (14) |  |
| Daughter | 2 (10) | 2 (15) | - |  |
| Sibling | 1 (5) | 0 (0) | - |  |
| Parent | 1 (5) | 1 (8) | - |  |
| Other | 1 (5) | 1 (8) | - |  |
| Cohabiting, n (%) | 13 (65) | 7 (54) | 6 (86) | 0.329^b^ |
| Education, n (%) |  |  |  | 0.029 ^b^ |
| Elementary/secondary | 10 (50) | 6 (46) | 4 (57) |  |
| University | 10 (50) | 7 (54) | 3 (43) |  |
| Working, n (%) | 6 (30) | 4 (31) | 2 (29) | 1.0^b^ |
| Economy, n (%) |  |  |  | 0.530 ^b^ |
| Insufficient | 0 (0) | 0 (0) | 0(0) |  |
| Just enough | 3 (15) | 2 (15) | 1 (14) |  |
| Good enough | 17 (85) | 11 (85) | 6 (86) |  |
| Received enough information to support significant other during hospital stay, n (%) |  |  |  |  |
| Yes | 6 (30) | 3 (23) | 3 (43) |  |
| Partly | 6 (30) | 6 (46) | 0 (0) |  |
| No | 7 (35) | 3 (23) | 4 (57) |  |
| Received enough information to support significant other after hospital stay, n (%) |  |  |  |  |
| Yes | 4 (20) | 2 (15) | 2 (29) |  |
| Partly | 8 (40) | 5 (39) | 3 (43) |  |
| No | 7 (35) | 5 (39) | 2 (29) |  |
| Assist in PADL, n (%) |  |  |  |  |
| Yes | 0 (0) | 0 (0) | 0 (0) |  |
| Sometimes | 2 (10) | 1 (8) | 1 (14) |  |
| No | 18 (90) | 12 (92) | 6 (86) |  |
| Assist in IADL, n (%) |  |  |  |  |
| Yes | 6 (30) | 3 (23) | 3 (43) |  |
| Sometimes | 5 (25) | 5 (39) | 0 (0) |  |
| No | 8 (40) | 4 (31) | 4 (57) |  |
| Assist with medications or contacts with healthcare, n (%) |  |  |  |  |
| Yes | 5 (25) | 2 (15) | 3 (43) |  |
| Sometimes | 5 (25) | 5 (39) | 0 (0) |  |
| No | 10 (50) | 6 (46) | 4 (57) |  |
| Assist with other, n (%) |  |  |  |  |
| Yes | 8 (40) | 7 (54) | 1 (14) |  |
| Sometimes | 0 (0) | 0 (0) | 0 (0) |  |
| No | 11 (55) | 5 (39) | 6 (86) |  |
| **Outcomes** |  |  |  |  |
| Caregiver burden, median (IQR) min-max | 10 (1-20) 0-38 | 10 (4-19) 0-25 | 8 (1-22) 1-38 | 0.780^a^ |
| Self-rated health, median (IQR) min-max | 76 (55-90) 20-100 | 72 (50-90) 20-100 | 80 (70-90) 70-90 | 0.395^a^ |
| Satisfied with life as a whole, n (%) | 10 (50) | 7 (54) | 3 (43) | 1.0^b^ |

Abbreviations: IQR= Interquartile range, PADL=Personal activities of daily living, IADL= Instrumental activities of daily living, ^a^= Mann Whitney U test ^b=^Fisher exact test
