# Supplementary material for: Feasibility, Fidelity and Acceptability of a Person‐Centred Care Transition Support Intervention for Stroke Survivors: A Non‐Randomised Controlled Study
Source: Health Expect. 2024 Oct 7;27(5):e70057. doi: 10.1111/hex.70057 (PMC11456962; doi:10.1111/hex.70057)
Supplement: Supplementary file 3 — Supporting information. [file HEX-27-e70057-s001.docx]

**Appendix 3.** The constructs, sub-constructs, related items and the average scores of 20 items of the Normalization Process Theory Measure.

|  |  | Item | Mean (SD) |
| --- | --- | --- | --- |
| *Coherence* | *Differentiation* | I can see how the [intervention] differs from usual ways of working | 3,8 (1,0) |
|  | *Communal specification* | Staff in this organisation have a shared understanding of the purpose of this [intervention] | 3,2 (0,9) |
|  | *Individual specification* | I understand how the [intervention] affects the nature of my own work | 4,2 (0,7) |
|  | *Internalization* | I can see the potential value of the [intervention] for my work | 4,2 (0,9) |
| *Cognitive Participation* | *Initiation* | There are key people who drive the [intervention] forward and get others involved | 4,4 (0,7) |
|  | *Legitimation* | I believe that participating in the [intervention] is a legitimate part of my role | 4,1 (0,8) |
|  | *Enrolment* | I’m open to working with colleagues in new ways to use the [intervention] | 4,5 (0,6) |
|  | *Activation* | I will continue to support the [intervention] | 4,4 (0,8) |
| *Collective Action* | *Interactional workability* | I can easily integrate the [intervention] into my existing work | 3,7 (0,9) |
|  | *Relational integration* | The [intervention] disrupts working relationships | 4,3 (0,8) |
|  | *Relational integration* | I have confidence in other people’s ability to use the [intervention] | 4,0 (0,7) |
|  | *Skill set workability* | Work is assigned to those with skills appropriate to the [intervention] | 3,7 (0,7) |
|  | *Skill set workability* | Sufficient training is provided to enable staff to use the [intervention] | 3,8 (0,8) |
|  | *Contextual Integration* | Sufficient resources are available to support the [intervention] | 3,1 (0,9) |
|  | *Contextual integration* | Management adequately support the [intervention] | 4,3 (0,7) |
| *Reflexive Monitoring* | *Systemisation* | I am aware of reports about the effects of the [intervention] | 2,4 (1,0) |
|  | *Communal appraisal* | The staff agree that the [intervention] is worthwhile | 3,3 (0,8) |
|  | *Individual appraisal* | I value the effects the [intervention] has had on my work | 3,7 (0,8) |
|  | *Reconfiguration* | Feedback about the [intervention] can be used to improve it in the future | 4,4 (0,7) |
|  | *Reconfiguration* | I can modify how I work with the [intervention] | 4,0 (0,7) |
