# Supplementary material for: Feasibility, Fidelity and Acceptability of a Person‐Centred Care Transition Support Intervention for Stroke Survivors: A Non‐Randomised Controlled Study
Source: Health Expect. 2024 Oct 7;27(5):e70057. doi: 10.1111/hex.70057 (PMC11456962; doi:10.1111/hex.70057)
Supplement: Supplementary file 4 — Supporting information. [file HEX-27-e70057-s005.docx]

**Appendix 4.** Comparison of primary and secondary outcome measures between intervention and control group at one week.

| **Variable** | **Value** | | | |
| --- | --- | --- | --- | --- |
|  | **Total, n=44** | **Intervention, n=25** | **Control, n=19** | **p-value** |
| **Primary outcome** |  |  |  |  |
| Perceived quality of care transition, mean (SD) min-max | 71 (25) 0-100 | 79 (19) 21-100 | 62 (29) 0-100 | 0.027^a^ |
| **Secondary outcomes** |  |  |  |  |
| Adherence to medical treatment |  |  |  |  |
| Mean (SD) | 24 (3.2) 5-25 | 23.6 (4.2) 5-25 | 24.5 (1.3) 20-25 | 0.349^a^ |
| Median (IQR) | 25 (25-25) 5-25 | 25 (24-25) 5-25 | 25 (25-25) 20-25 | 0.321^b^ |
| Health literacy |  |  |  |  |
| Subscale 1: Feeling understood and supported by healthcare providers | 2.8 (0.7) 2.6-3.1 | 2.9 (0.8) 2.6-3.3 | 2.7 (0.7) 2.4-3.1 | 0.382^a^ |
| Subscale 2: Having sufficient information to manage my health | 2.9 (0.6) 2.7-3.1 | 2.9 (0.6) 2.7-3.2 | 2.8 (0.6) 2.5-3.1 | 0.377^a^ |
| Subscale 3: Actively managing my health | 2.7 (0.6) 2.6-2.9 | 2.8 (0.6) 2.6-3.1 | 2.7 (0.6) 2.4-3.0 | 0.435^a^ |
| Subscale 4: Social support for health | 3.2 (0.5) 3.0-3.3 | 3.2 (0.5) 3.0-3.4 | 3.2 (0.5) 2.9-3.4 | 0.889^a^ |
| Subscale 5: Appraisal of health information | 2.4 (0.6) 2.2-2.6 | 2.5 (0.6) 2.2-2.7 | 2.3 (0.6) 2.0-2.6 | 0.442^a^ |
| Subscale 6: Ability to actively engage with healthcare providers | 3.7 (0.8) 3.5-4.0 | 3.8 (0.9) 3.4-4.2 | 3.6 (0.8) 3.2-4.0 | 0.417^a^ |
| Subscale 7: Navigating the healthcare system | 3.5 (0.9) 3.3-3.8 | 3.6 (0.9) 3.3-4.0 | 3.4 (0.8) 3.0-3.8 | 0.351^a^ |
| Subscale 8: Ability to find good health information | 3.6 (0.9) 3.3-3.9 | 3.7 (0.9) 3.7-4.1 | 3.5 (0.9) 3.0-3.9 | 0.429^a^ |
| Subscale 9: Understanding health information well enough to know what to do | 3.9 (0.8) 3.7-4.2 | 4.0 (0.9) 3.6-4.4 | 3.8 (0.7) 3.5-4.2 | 0.433^a^ |
| Self-rated recovery |  |  |  |  |
| Mean (SD) min-max | 74 (21) 18-100 | 73 (24) 18-100 | 74 (17) 40-99 | 0.896^a^ |
| Median (IQR) min-max | 80 (63-90) 18-100 | 80 (63-90) 18-100 | 79 (63-89) 40-99 | 0.687^b^ |
| Knowledge of new  Medication after discharge, n (%) (Missing 7) | 34 (69) | 21 (75) | 13 (62) | 0.112^c^ |
| Knowledge of changes in  Medication, n (%) (Missing 7) | 34 (69) | 18 (64) | 16 (76) | 0.709^c^ |

Abbreviations: SD=Standard deviation, IQR= Interquartile range.  ^a^Students T-Test ^b^Mann Whitney U test, ^c^Fisher exact test.
