# Supplementary material for: Feasibility, Fidelity and Acceptability of a Person‐Centred Care Transition Support Intervention for Stroke Survivors: A Non‐Randomised Controlled Study
Source: Health Expect. 2024 Oct 7;27(5):e70057. doi: 10.1111/hex.70057 (PMC11456962; doi:10.1111/hex.70057)
Supplement: Supplementary file 5 — Supporting information. [file HEX-27-e70057-s003.docx]

**Appendix 5.** Comparison of characteristics and outcomes at 3 months between intervention and control group.

| **Variable** | **Value** | | | |
| --- | --- | --- | --- | --- |
|  | **Total, n=41** | **Intervention, n=23** | **Control, n=18** | **p-value** |
| Barthel Index |  |  |  |  |
| Mean (SD) | 98 (6) 75-100 | 96.7 (7.5) 75-100 | 98.6 (3.8) 85-100 | 0.327^a^ |
| Median (IQR) | 100 (100-100) 75-100 | 100 (99-100) 75-100 | 100 (100-100) 85-100 | 0.435^b^ |
| Fatigue | 40 (29) 0-100 | 35 (26.9) 0-80 | 45 (31) 0-100 | 0.383^a^ |
| Disability, n (%) |  |  |  | 0.487^c^ |
| Mild | 29 (59) | 14 (50) | 15 (71) |  |
| Moderate | 11 (22) | 7 (25) | 4 (19) |  |
| Severe | 2 (4) | 2 (7) | 0 (0) |  |
| Signs of cognitive impairment, n (%) | 13 (27) | 6 (21) | 7 (33) | 0.520^c^ |
| Signs of depression, n (%) | 8 (16) | 4 (14) | 4 (19) | 1.0^c^ |
| Adherence to medical treatment |  |  |  |  |
| Mean (SD) | 24 (2) 13-25 | 24 (2.6) 13-25 | 24.8 (0.5) 23-25 | 0.199^a^ |
| Median (IQR) | 25 (24-25) 13-25 | 25 (24-25) 13-25 | 25 (25-25) 23-25 | 0.134^b^ |
| Health literacy |  |  |  |  |
| Subscale 1: Feeling understood and supported by healthcare providers | 3.1 (0.7) 2.9-3.3 | 2.9 (0.8) 2.6-3.3 | 3.2 (0.4) 3.0-3.4 | 0.180^a^ |
| Subscale 2: Having sufficient information to manage my health | 2.9 (0.6) 2.8-3.1 | 3.0 (0.6) 2.8-3.3 | 2.9 (0.5) 2.7-3.2 | 0.558^a^ |
| Subscale 3: Actively managing my health | 2.9 (0.5) 2.7-3.0 | 2.9 (0.6) 2.6-3.1 | 2.9 (0.5) 2.6-3.1 | 0.947^a^ |
| Subscale 4: Social support for health | 3.3 (0.6) 3.1-3.4 | 3.1 (0.6) 2.8-3.4 | 3.4 (0.4) 3.2-3.6 | 0.132^a^ |
| Subscale 5: Appraisal of health information | 2.5 (0.6) 2.4-2.7 | 2.6 (0.6) 2.3-2.9 | 2.4 (0.6) 2.2-2.7 | 0.404^a^ |
| Subscale 6: Ability to actively engage with healthcare providers | 3.9 (0.9) 3.6-4.2 | 3.8 (1.1) 3.3-4.3 | 3.9 (0.8) 3.5-4.3 | 0.773^a^ |
| Subscale 7: Navigating the healthcare system | 3.6 (0.9) 3.3-3.9 | 3.6 (1.0) 3.2-4.1 | 3.7 (0.8) 3.3-4.1 | 0.919^a^ |
| Subscale 8: Ability to find good health information | 3.6 (1.0) 3.3-4.0 | 3.6 (1.1) 3.1-4.1 | 3.7 (0.8) 3.3-4.1 | 0.769^a^ |
| Subscale 9: Understanding health information well enough to know what to do | 4.0 (0.8) 3.8-4.3 | 4.0 (0.9) 3.6-4.4 | 4.1 (0.7) 3.8-4.4 | 0.718^a^ |
| General self-efficacy | 33.2 (5.1) 20-40 | 34.1 (5.7) 20-40 | 32.2 (4.2) 25-39 | 0.234^a^ |
| Self-rated recovery |  |  |  |  |
| Mean (SD) min-max | 85 (14.7) 50-100 | 83 (16.3) 50-100 | 88 (12.6) 60-100 | 0.356^a^ |
| Median (IQR) min-max | 90 (80-96) 50-100 | 90 (70-95) 50-100 | 90 (80-98) 60-100 | 0.453^b^ |
| Knowledge of new  medication after discharge, 1 week, n (%) | 24 (49) | 12 (43) | 12 (57) | 0.752^c^ |
| Knowledge of changes in medication, 1 week, n (%) | 33 (67) | 20 (71) | 13 (62) | 0.115^c^ |

Abbreviations: Abbreviations: SD=Standard deviation, IQR= Interquartile range.  ^a^Students T-Test ^b^Mann Whitney U test, ^c^Fisher exact test.
